# Advanced integrated strategy for structural and mineralogical exploration of inaccessible regions employing remote sensing and multiscale analysis of aeromagnetic data

**DOI:** 10.1038/s41598-025-16618-w

**Published:** 2025-08-25

**Authors:** Ahmed M. Eldosouky, Mohamed A. Abd El-Wahed, Mohamed Attia, Saada A. Saada, Mahmoud Ahmed Abbas

**Affiliations:** 1 Department of Geology, Faculty of Science, Suez University, P.O. Box: 43221, Suez, Egypt; 2 Geology Department, Faculty of Science, Tanta University, P.O. Box: 31527, Tanta, Egypt; 3 State Key Laboratory of Continental Dynamics, Department of Geology, Northwest University, Xi’an, 710069 China; 4 Geology Department, Faculty of Science, Kafr El Sheikh University, P.O. Box: 33511, Kafr El Sheikh, Egypt; 5 Department of Earth, Environmental and Resources Sciences (DiSTAR), University of Naples Federico II, Naples, 80126 Italy; 6 Geology Department, Faculty of Science, South Valley University, 83523 Qena, Egypt

**Keywords:** Tectonics, Aeromagnetic, Edge detection, Geophysics, Remote sensing, Mineralization, Eastern desert., Solid Earth sciences, Geology, Geophysics

## Abstract

Rugged terrains and remote desert environments present notable challenges for geological data analyses due to limited accessibility and scarcity of surface and subsurface data. One of such challenging areas is Wadi Dif, located in South Eastern Desert (SED) of Egypt. This study presents an integrated approach combining aeromagnetic and remote sensing data to effectively investigate such environments. A Multiscale Derivative Analysis (MDA), utilizing the Enhanced Horizontal Derivative (EHD)of, is applied to reduced-to-the-pole aeromagnetic data of Wadi Dif area. This method is formed by a weighted sum of increasing order derivatives of the field data and enables high-resolution delineation of both deep-seated and shallow geologic structures. Additionally, color composites imagery derived from remotely sensed data played a vital role in lithological and structural mapping. The obtained results from remote sensing and geophysical observations for shallow and deep structures were used to outline the deformation history of Wadi Dif area. This deformation history begins with early NNE-SSW crustal shortening, followed by NNW-SSE folds and crenulation cleavage in phase D2. Crenulations and kink folds emerge from oblique non-coaxial deformation of cleaved rocks. The Kharit graben and Cretaceous sediments are formed in phase D4, followed by ENE-WSW dextral and N-S sinistral strike-slip faults that further alter preexisting rocks and displaced earlier structures. The distribution of lineament density and surface alteration zones yielded two maps highlighting areas with possible ore deposits. Alteration zones which are mainly propylitic zones, CO3 and Mg-OH bearing minerals are associated with areas of moderate to high lineament density, which facilitated fluid movement. However, not all high-density areas showed alteration, likely due to differing rock composition. Lineament trends mainly follow N-S and NW directions, aligning with the Hamisana shear zone and Najd fault system, suggesting they are pathways for ore fluids. The integration of MDA of aeromagnetic data with remote sensing data improves structural interpretation and mineral potential appraisal in inaccessible regions where traditional fieldwork is inoperable like Wadi Dif area. This approach proves effective in delineating fault systems, geological boundaries, and deformation patterns, presenting an invaluable tool for mapping deep-seated and shallow structures and mineral potentials in arid remote environments.

## Introduction

Rugged and harsh desert terrains create major challenges for geological investigation due to their extreme climatic conditions, challenging geography, and logistical limitations. Consequently, the hardship of operating ground surveys in such regions influences a lack of high-resolution geological, mineralogical, and structural data, resulting in significant knowledge gaps in detailed and regional geological analyses.

To overcome these limitations, recent advances in multi-sensor satellites including Landsat, ASTER, Sentinel and Radar platforms have proven to be valuable tools for lithological and structural analysis or mapping^1–7^. Specifically, their VNIR and SWIR spectral bands as well as the microwaves can discriminate between the different rock units, identify hydrothermal alteration zones, trace and extract major structural elements and lineaments (e.g., faults, fractures, joints and folds)^3,5,7–16^.

In addition to satellite data, potential fields methods like gravity and magnetic surveys play an essential part in mapping the structures by providing a high-resolution understanding of subsurface geologic characteristics^17–22^. For example, analyzing aeromagnetic data, fault structures, folding systems, and rock boundaries, presents more profound insights into the area’s tectonic pattern^23–26^. The use of advanced edge detectors further improves structural understanding by determining the directions and locations of geologic discontinuities, assembling them as essential techniques for unraveling deformation systems and estimating crustal architecture^27–35^. On a broader scale, aeromagnetic anomalies help regional tectono-structural investigations by revealing deep-seated systems that control magmatic intrusions, basin evolution, and fault patterns. This information is quite invaluable for reconstructing geodynamic records and comprehending crustal deformation processes^14,36–39^. Furthermore, aeromagnetic analyses are widely employed in mineral potential investigation because they help identify structural traps, igneous intrusions, and pathways of hydrothermal solutions that can host considerable mineralization^14,15,40–49^.

The integration of airborne geophysical data with remote sensing (RS) and geological datasets can enhance the efficiency of exploration and structural delineation. This integrated approach helps identify target sites and support resource appraisals, especially in complicated and rugged environments^14,15,41,48,50–53^.

The Wadi Dif area, located in the southern Eastern Desert of Egypt, represents such a challenging environment. It is a remote mountainous region characterized by Phanerozoic sedimentary depositsunconformably overlying Precambrian bedrock. The Precambrian rocks mainly consist of granitic formations that have intruded surrounding units, forming thermal aureoles at contact zones with metavolcanic materials. The area’s sedimentary cover belongs to the Kharit basin, part of the South Egypt rift system which is characterized as an extended sedimentary inlier orientated northwest, located inside the crystalline bedrock of the Arabian-Nubian shield^54^. The Kharit basin has northwest-striking, fault-bounded half-grabens linked to northeast-dipping syn-rift deposits. The rift’s geometry is affected by the reactivation of a Precambrian Najd-parallel shear zone positioned at around 125°E at the onset of the rifting process^55^ .

Building on these insights, our study aims to apply a refined, integrated approach combining aeromagnetic and RS data to accurately delineate geological structures in remote and rugged desert areas like Wadi Dif, South Eastern Desert (SED), Egypt. Specifically, we will apply Multiscale Derivative Analysis (MDA) to aeromagnetic data to accurately identify shallow and deep-seated structures. Complementary RS image analyses will support detailed mapping of lithology, lineaments, and alteration zones. By integrating the outputs, the study seeks to provide a comprehensive understanding of subsurface structural patterns and assess the mineral possibility of these challenging terrains. The main objective is to present a cost-effective, efficient, and reliable procedure for geological investigation in regions of remote desert environments. Furthermore, the employing of both data types along with the field survey to identify the alteration zones which are considered one of the favorable sites for ore deposits such as gold, copper and iron oxides creating a potential map for these ores. The integration among the multi-data types (e.g., Rs, MDA and field data) not only helped in the explorations of mineralization areas but also has been utilized as a vital tool to investigate the major surface and subsurface structural elements to decipher and rebuild the tectonic senior for such hard-to-reach areas including our presented area.

## Geological setting

**Fig. 1:**
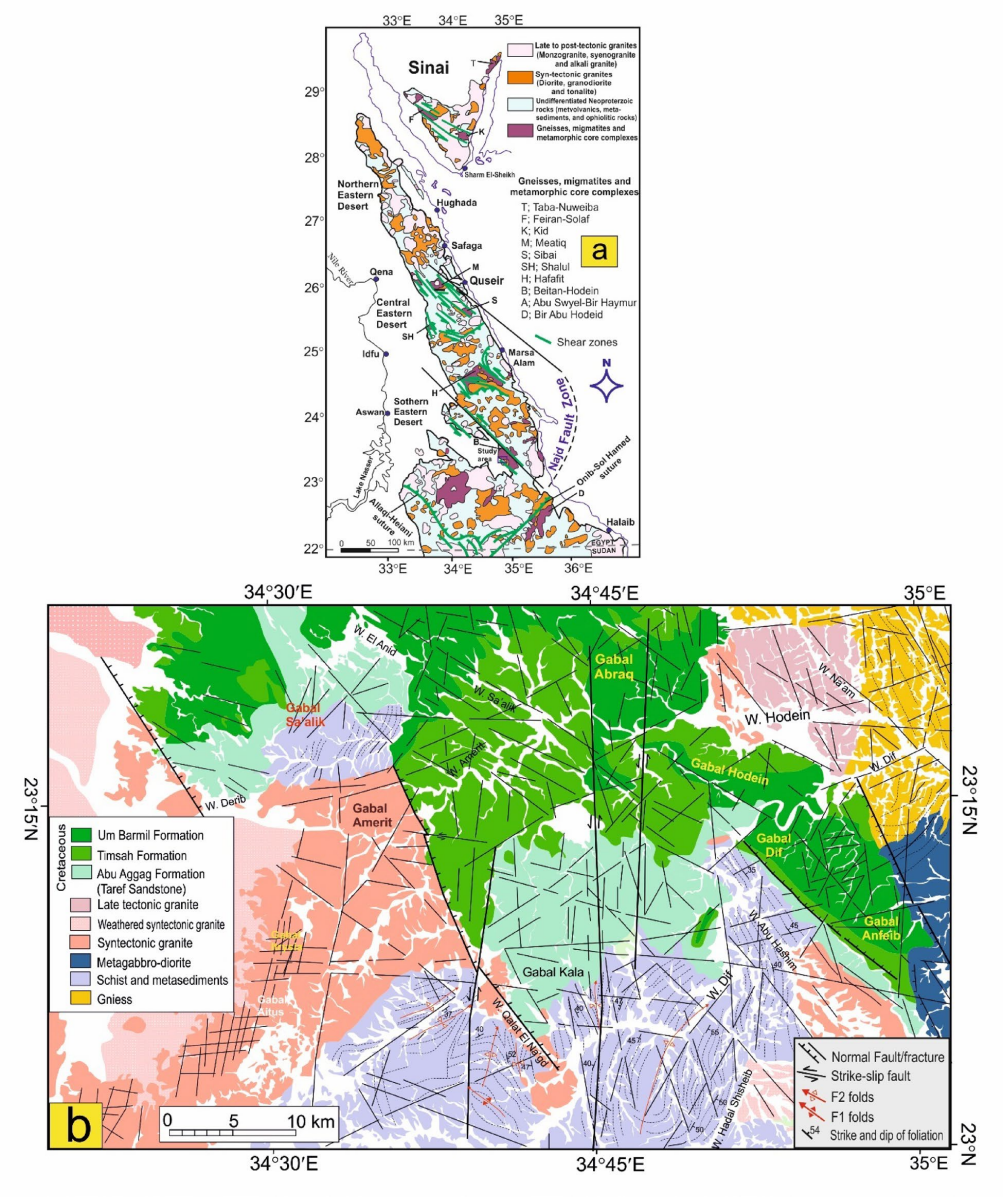
**a**) Simplified geologic map of the Eastern Desert, Egypt, (after Zoheir et al.^11^; compilation by A.-R. Fowler cited in Johnson et al.^65^ showing the area affected by the Najd Fault System in the Egyptian Nubian Shield including the study area, **b**) Geological map of Wadi Dif area (modified and complied from Ashmawy^56^ and Conoco^66^. (The figure was created by ArcGIS Desktop v 10.8. https://www.esri.com/en-us/arcgis/products/arcgis-desktop/overview, and SmartSketch v. 4.0 software; https://smartsketch.software.informer.com/4.0/).

The gneissic rocks, located in the northeastern corner of the study area (Figs. 1a and b), are part of the Beitan-Hodein gneiss complex, constituting a prominent NW-trending belt composed of a variety of gneisses, including fine to coarse-grained biotite gneisses, biotite-hornblende gneisses, and hornblende gneisses, alongside lesser amounts of garnet-biotite-hornblende gneisses, augen gneiss, amphibolites, migmatites, and mylonites^4,56–62^. Gneisses are distinguished by their banded appearance and isoclinal folding, while migmatites exhibit ptygmatic and nebulitic structural features. The ptygmatic migmatites exhibit highly disharmonic, convolute, and tortuous folding patterns. Zircon analyses from the Wadi Beitan gneisses have provided a mean Pb–Pb evaporation age of 704 ± 8 million years^63^. Additionally, Sm–Nd isotopic dating has yielded ages ranging from 744 ± 9 to 719 ± 10 million years for the Beitan gneisses, while magmatic zircons have produced mean 206Pb/238U ages of 744 ± 10, 725 ± 9, and 719 ± 10 million years^64^.

Schists are prominently exposed as moderate relief hills in the southern region (Fig. 1b) of the study area. This geological unit includes tourmaline-almandine-staurolite-biotite schist and almandine-hornblende-biotite schist^57^. These schists exhibit a moderate to weak schistose texture and are intruded by late tectonic granite, with faulting occurring against Cretaceous sediments. The metagabbro-diorite rocks display a range of deformation, from massive forms to those that are strongly sheared or foliated. The interface between metagabbro diorite and gneisses is marked by prominently foliated diorite and amphibolite^58^. Additionally, these rocks are penetrated by syntectonic to late tectonic granites that are moderately to highly weathered. The syntectonic granite includes quartz diorite, tonalite, and granodiorite. Weathering phenomena such as exfoliation, tafone, and spheroidal boulders are frequently observed in the syntectonic outcrops. These rocks are predominantly medium- to coarse-grained, exhibiting colors ranging from medium gray to pinkish-gray and light gray. The boundaries between late tectonic granite and surrounding country rocks are typically sharp and steeply inclined outward from the granite plutons. The late tectonic granites are generally homogeneous, lacking internal structure, and are commonly medium- to coarse-grained, displaying pale red, red, rosy, and grayish-pink hues^57^.

The study area is covered mainly by Phanerozoic sedimentary rocks set unconformably over basement rocks of Precambrian age (Fig. 1b). The Precambrian units are occurring as highly fractured hills of gneissic granite and younger granite of tonalitic to granodioritic composition intruded into the surrounding rocks, forming thermal aureoles along the contacts with these rocks as well as some metavolcanics. The Cretaceous sediments in the Wadi Dif area are part of the Kharit basin. This basin is the southern segment of the South Egypt rift system and appears as an elongated NW-oriented sedimentary inliers within crystalline basement rocks of the Arabian -Nubian shield^54^. The Kharit basin is a NW-striking fault-bounded half-grabens associated with NE-dipping syn-rift rocks. This rift geometry is controlled by the reactivation of a Precambrian Najd-parallel (N125°E) shear zone during rift initiation^55^.

From the oldest to the youngest, the study area comprises the following Nubia succession: they are Abu Aggag Formation, Timsah Formation, and Umm Brammily Formation (Fig. 1b), which are set unconformably on the Precambrian assemblage^67,68^. The Abu Aggag Formation is a large-scale fining-upward cycle of braided stream systems, consisting of three successive units: a basal massive kaolinitic conglomerate unit, a middle trough and tabular cross-bedded conglomerate and conglomeratic sandstone unit, and an upper mudstone-dominated unit. The formation is barren of fossils but may contain plant remains and root molds^69^. The Timsah Formation is composed of sandstone and tough clays. The middle unit of the coarsening-upward cycle is composed of rhythmically alternating beds of kaolinitic, oolitic ironstone, and mudstone^70^. The Um Brammily Formation is composed of fluviatile sandstone, deposited during sea retreat during the Santonian-Campanian time^67,70^.

## Methodology

We implement an integrated approach combining aeromagnetic data analysis with remote sensing (RS) techniques, supported by field observations, to effectively delineate geological structures and assess mineralization potential in remote desert terrains, as in the Wadi Dif area, South Eastern Desert (SED), Egypt.

### Magnetic data

We apply a Multiscale Derivative Analysis (MDA) to aeromagnetic data, enabling the detection of both shallow and deep-seated subsurface structures.

#### Multiscale derivative analysis

Accurately delineating the horizontal boundaries of potential field sources is critical for interpreting the structural framework related to mineralization. Nevertheless, advanced techniques are required to obtain reliable results, such as the Multiscale Derivative Analysis (MDA)^71^which is based on the Enhanced Horizontal Derivative (EHD) method, introduced by Fedi and Florio^72^. The enhanced horizontal derivative (EHD) method is a high-resolution method designed to delineate the horizontal boundaries of potential field causative sources. It operates by calculating the horizontal derivative of a sum of vertical derivatives of increasing order of the potential field (gravity or magnetic data). The maxima of this derivative function is then used to outline source edges. The EHD method can be applied to reduced-to-the-pole magnetic anomalies^72^.

This approach was successfully implemented in various recent studies, including the identification of gravimetric lineaments for faults characterization^73^mapping the lateral boundaries of magnetic anomalies sources associated archeological bodies^74^and the delineation of the boundaries of salt structures^34^.

To formalize this method, the following equations define the EHD in Cartesian coordinates (*x*, *y*). The EHD of the magnetic field map *f* (*x*,*y*) is given by^72^:

1 $$\:EHD\left(x,y\right)=\sqrt{\frac{\partial\:\phi\:}{\partial\:x}+\frac{\partial\:\phi\:}{\partial\:y}}$$

where,

2 $$\:\phi\:\left(x,y,z\right)=f\left(x,y,z\right)+{w}_{1}{f}_{1}\left(x,y,\:{z}_{0}\right)+{w}_{2}{f}_{2}\left(x,y,\:{z}_{0}\right)+\dots\:+{w}_{k}{f}_{k}\left(x,y,\:{z}_{0}\right)$$

In this formulation, *f*_*k*_ is the *k*^th^ vertical derivative of *f*, and the weights *w*_*k*_ are defined as*w*_*k*_=*q*^*k*^, with *q* is an appropriate constant with length dimension.

The maxima of the EHD, computed from either gravity anomalies or pole-reduced magnetic anomalies, effectively trace the boundaries of the causative source. By selecting appropriate derivative orders and weights, the method can be adapted to produce EHD maps at various scales: higher order derivatives emphasize small-scale, shallow sources, whereas the lower orders highlight large-scale, deeper sources.

Consequently, the magnetic data analysis is complemented by advanced RS image processing for lithological discrimination, lineament extraction, and identification of hydrothermal alteration zones which indicate the potential areas of mineralization.

### Remotely sensed datasets

Cloud-free optical datasets, including Landsat-8L1TP (acquired on 19th of Jan. 2025, path 173/row 44) and four ASTER L1T scenes (acquired on 3rd of Aug. 2001). Moreover, Sentinel-1B (S1B) radar data Level-1 Ground Range Detected (GRD) (acquired on 23rd of Dec. 2023), and two DEM scenes (acquired on 6th of Aug. 2015) were obtained for the presented area. Both optical data were employed for the lithological, alteration zones and structural mapping, while the S1B radar data was utilized for the extraction of lineaments automatically. Additionally, the elevation data (DEM) was employed to create a hill-shaded map for the study area. Eleven spectral bands including panchromatic, VNIR, SWIR and thermal bands of different spatial resolutions that vary from 15 to 90 m are characterized Landsat-8 (Table 1), while the ASTER are marked by fourteen spectral bands (e.g., VNIR, SWIR, thermal bands, Table 1) of variable spatial (15 and 30 m) and spectral resolutions with the first nine bands particularly effective for detecting alteration minerals associated with ore deposits. C-bands synthetic aperture radar (SAR) of S1B (GRD) are distinguished by single polarization (VH, VV) for the Wave mode and dual polarization (VV + VH) for the other different modes and have different spatial resolutions and swaths up to 400 km-wide (Table 2).

**Table 1:** The defining features of spectral ranges and the Spatial resolution pertinent to optical data.

Landsat-8 OLI				ASTER			
Bands (B)	Wavelength (µm)	Spectral Region	Resolution (m)	Bands (B)	Wavelength (µm)	Spectral Region	Resolution (m)
B2	0.452–0.512	B	30	B1	0.52–0.60	VNIR	15
B3	0.533–0.590	G		B2	0.63–0.69		
B4	0.636–0.673	R		B3	0.63–0.69		
B5	0.851–0.879	NIR		B4	1.60–1.70	SWIR	30
B6	1.566–1.651	SWIR		B5	2.145–2.185		
B7	2.107–2.294	SWIR		B6	2.185–2.225		
				B7	2.235–2.285		
-	-	-	-	B8	2.295–2.365		
-	-	-	-	B9	2.360–2.430		

Abbreviation: Blue = B; Green = G; Red = R; Near Infrared = NIR; Short wave Infrared = SWIR; Visible Nera Infrared = VNIR.

**Table 2:** Attributes of the Sentinel-1B radar data.

Radar AM	Sentinel-1			
	Stripmap (SM)	Interferometric wide swath (IW)	Extra wide swath (EW)	Wave (WV)
Beam Mode	S1 to S6	IW1 to IW3	EW1to EW5	WV1&WV2
Center Frequency	C-band (5.405 GHz)			
Polarization	SP (HH or VV) DP (HH + HV and VV + VH)			SP (HH or VV)
Spatial Resolution For S1B: (range x azimuth), m x m	5х5 m	5х20 m	25х100 m	5х20 m
Swath/band Width Km	80 km	250 km	4 km	20х20 Km
Off-Nadir Angle	-	-	-	-
Chirp Bandwidth [MHz]	87.6–42.2 MHz	56.5–42.8 MHz	22.2–10.4 MHz	74.5 & 48.2 MHz
Incidence Angle (deg)	20–43^◦^	30–42^◦^	20–44^◦^	23 & 36.5^◦^

Abbreviation: AM = Acquisition mode Single Polarization = SP, Daul Polarization = DP, Degree = deg.

### Technical peparation and processing of satellite data

Optical bands ranging from 2 to 7 of Landsat-8, along with bands 1 to 9 from ASTER, as well as S1B radar and DEM datasets were layer-stacked, mosaicked, subsetted and processed using ENVI5.3 (https://www.l3harrisgeospatial.com/Software-Technology/ENVI), Arc-Map-10.8 (https://desktop.arcgis.com/en/arcmap/index.html), PCI geomatica-2016 https://www.perspectivegeomatics.com/pci_geomatica and RockWork 16.

https://www.rockware.com/product/rockworks/ software to emphasize the area under examination and facilitate each of the lithological discrimination, alteration mapping, and the extraction of lineaments.

The preprocessing of the satellite data is includes: i) geometrically reprojection of the optical, radar, and DEM data to the UTM projection (Universal Transverse Mercator), Zone N36, utilizing the WGS-84 datum system; ii)for the atmospheric effects, cross-track illumination was used for Landsat-8 OLI, while iii) the Internal Average Relative Reflection (IARR) algorithm was applied on ASTER to mitigate atmospheric influences and transform the radiance data into surface reflectance data. Based on the filters supplemented in ENVI, the Enhanced Lee filter was implemented on the S1B radar data to minimize and eliminate speckles, while the DEM data was pre-processed using ArcMap 10.8 by applying the Filling order within the spatial analysis tools in ArcMap.

For the processing, various image transformations, such as False Color Composite (FCC), Band Ratios (BRs), Decorrelation Stretch (DS), and Principal Component Analysis (PCA), along with the constrained energy minimization (CEM) technique, were utilized and employed during the processing phase of the optical satellite datasets.

Numerous researchers^5–9,11–15,75^ have acknowledged that the precise lithological and structural mapping as well as the identification of hydrothermal alteration zones in greyscale or RGB mode can be effectively achieved through the application of BRs and PCA. These transformation techniques are recognized for capturing the highest data variability within the different color modes, particularly in the initial three principal components (PC1, PC2, PC3). Campbell and Wynne^76^ indicated that the selection of band combinations in RGB mode, based on their spectral characteristics, can be utilized to achieve optimal differentiation of rock units while simultaneously emphasizing significant geological structures and zones of hydrothermal alteration. Consequently, the tonal variations observed in the resultant-colored images facilitate the visual interpretation of lithological distinctions, regional structural features (such as strike-slip faults, thrusts, dykes, and joints), as well as hydrothermal alteration zones, which are subsequently validated through field investigations. In addition to the image transformations, the CEM technique attached ENVI spectral tool was utilized to identify alteration minerals and their spatial distribution in specified region. This analysis was based on the diagnostic spectral characteristics of each mineral, as documented in the accompanying spectral library of the Advanced Spaceborne Thermal Emission and Reflection Radiometer (ASTER).

The S1B radar data underwent Band Math (BM) processing, resulting in the combination of VV and VH polarizations into a composite layer (VV + VH), which was subsequently stacked with the individual polarizations (VV and VH). Following this, PCA was conducted on the stacked layers (VV, VH, and VV + VH), yielding three principal components (PC1, PC2, and PC3). The first principal component (PC1) was then processed using the LINE algorithm function integrated within PCI Geomatica software, allowing the automated extraction of structural elements, specifically lineaments. This extraction was guided by several parameters, including a filter radius of 10, a gradient threshold of 100, a length threshold of 30, a fitting threshold of 3, an angular threshold of 15, and a distance threshold of 20.

### Structural analysis

Structural analysis employs satellite imagery, along with magnetic datasets, to characterize geological structures identified in the research. This approach facilitates the development of tools and methodologies that allow for the identification of structures that are too extensive to be directly observed. Such structures encompass bedding planes, foliation planes, dykes, sills, fractures, faults, and folds.

Finally, the integration of aeromagnetic, RS, and ground truth data contributes to a more comprehensive understanding of the tectonic evolution and geological setting of the study area.

## Results

The subsequent sections present the results from the aeromagnetic analysis, RS interpretation, and field observations, emphasizing how the integration of these techniques enhances the interpretation of both surface and subsurface geological features.

### Aeromagnetic analysis

**Fig. 2:**
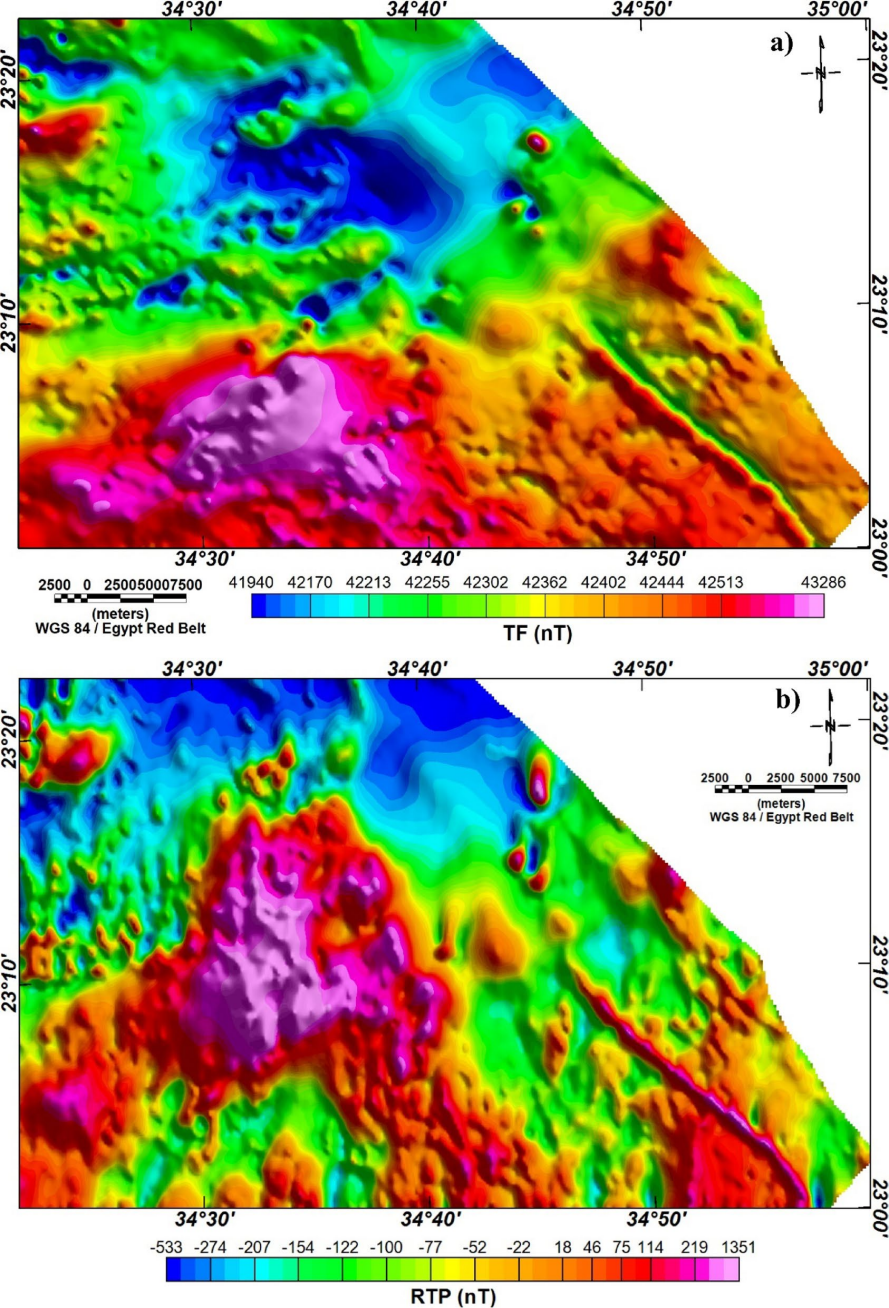
**a**) TMF map; and **b**) RTP map of Wadi Dif area. (The figure was created by Geosoft Oasis montaj v. 8.3.3. https://www.seequent.com/products-solutions/oasis-montaj/).

The total magnetic field data (TMF) of Wadi Dif area^77^shown in Fig. 2a are reduced-to-the-pole (RTP) to locate the ridges of anomalies over their magnetic sources^78^ (Fig. 2b). RTP map (Fig. 2b) shows magnetic anomaly field variations from low (-533 nT) to high (1351 nT) values. Low magnetic values are mainly related to areas with sedimentary cover while the magnetic anomaly values increase over basement rocks.

In the present work, we applied MDA technique to RTP map and its vertical derivatives from the 1st to the 4th order of differentiation. Different values of *q* are tested until we found an optimal definition of lineaments with *q* equal to 1 and 5 for shallow- or small- and regional- or large-scale structures, respectively.

**Fig. 3:**
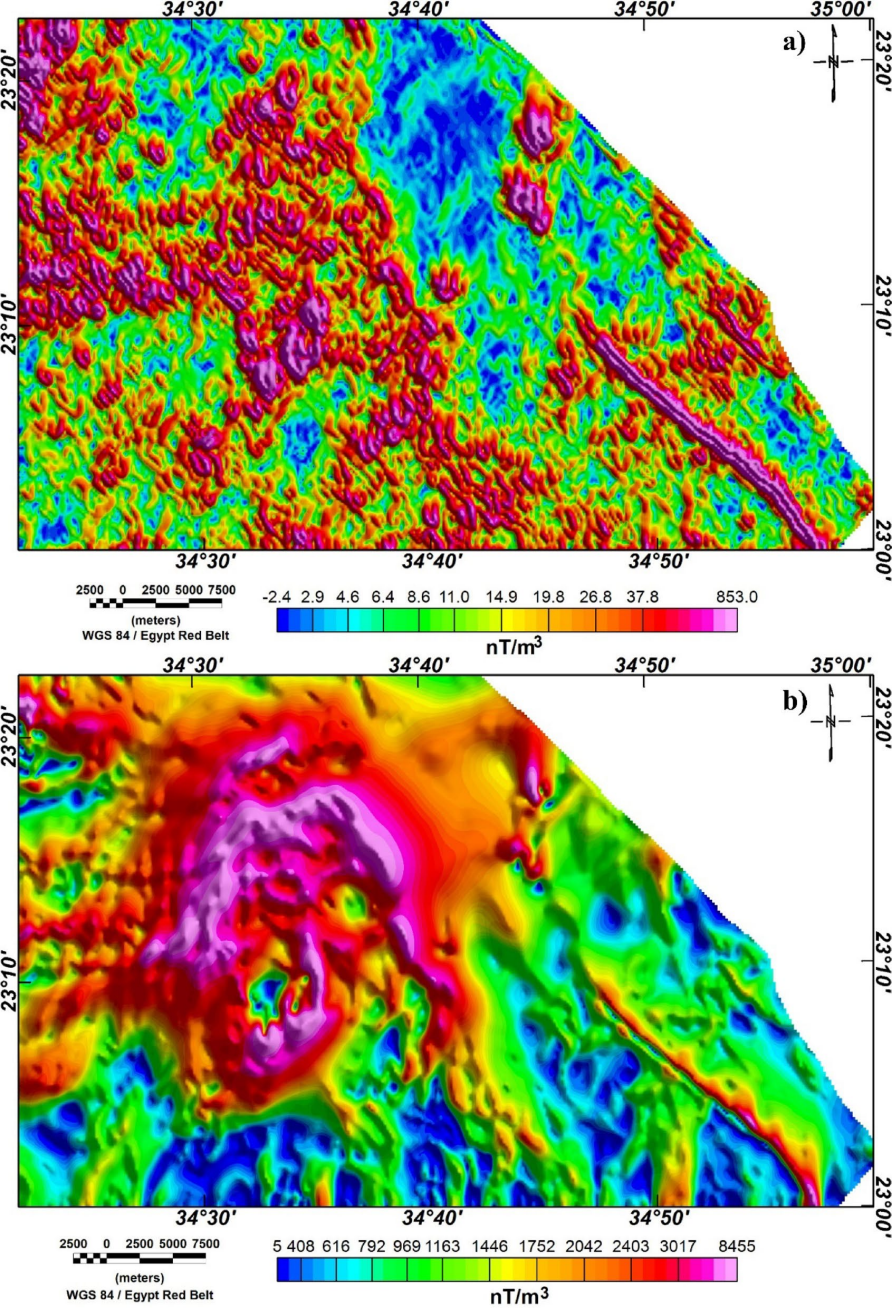
**a**) Small-scale (shallow) MDA-EHD map; and **b**) Large-scale (regional) map. (The figure was created by Geosoft Oasis montaj v. 8.3.3. https://www.seequent.com/products-solutions/oasis-montaj/).

**Fig. 4:**
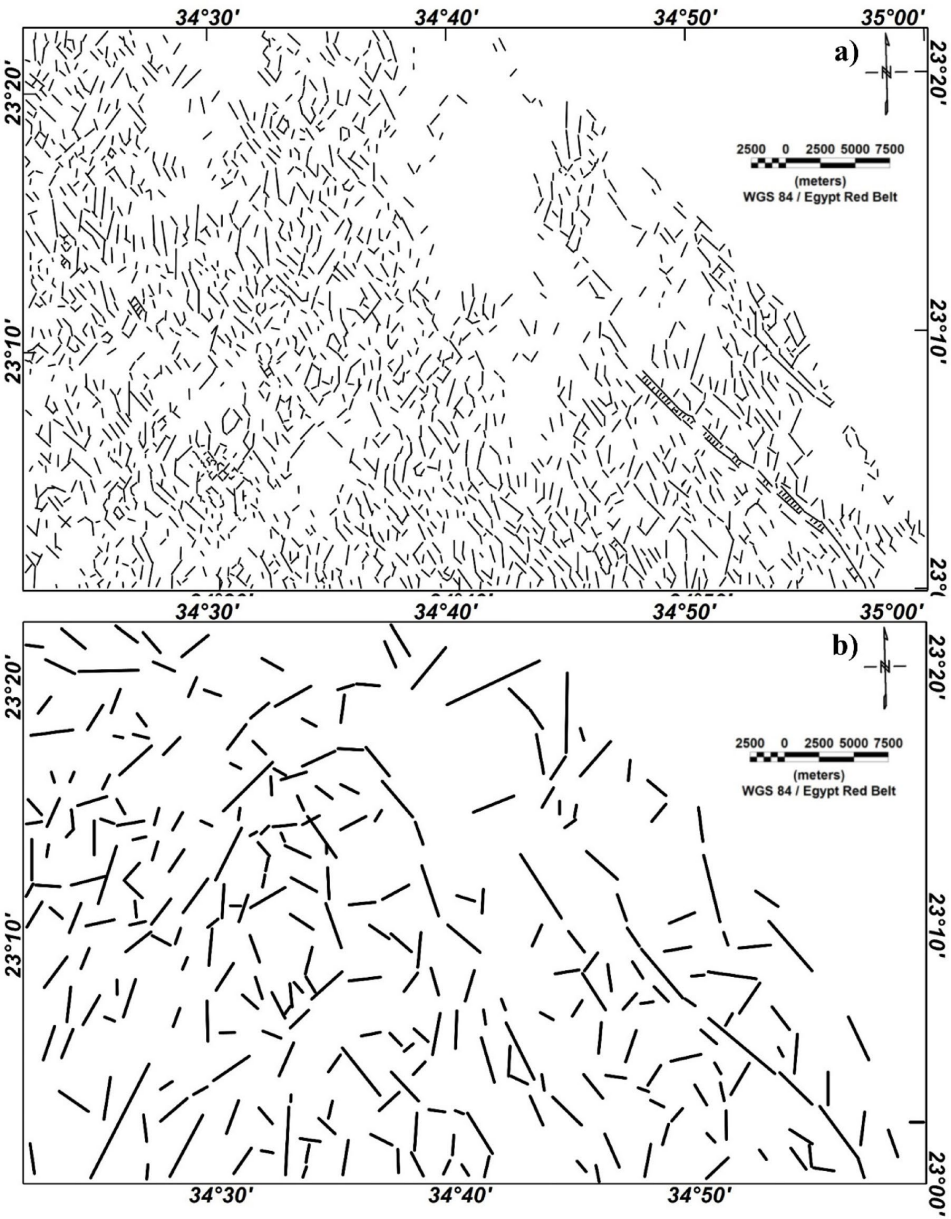
**a**) Small-scale (shallow) MDA-EHD lineaments; and **b**) Large-scale (regional) lineaments.

**Fig. 5:**
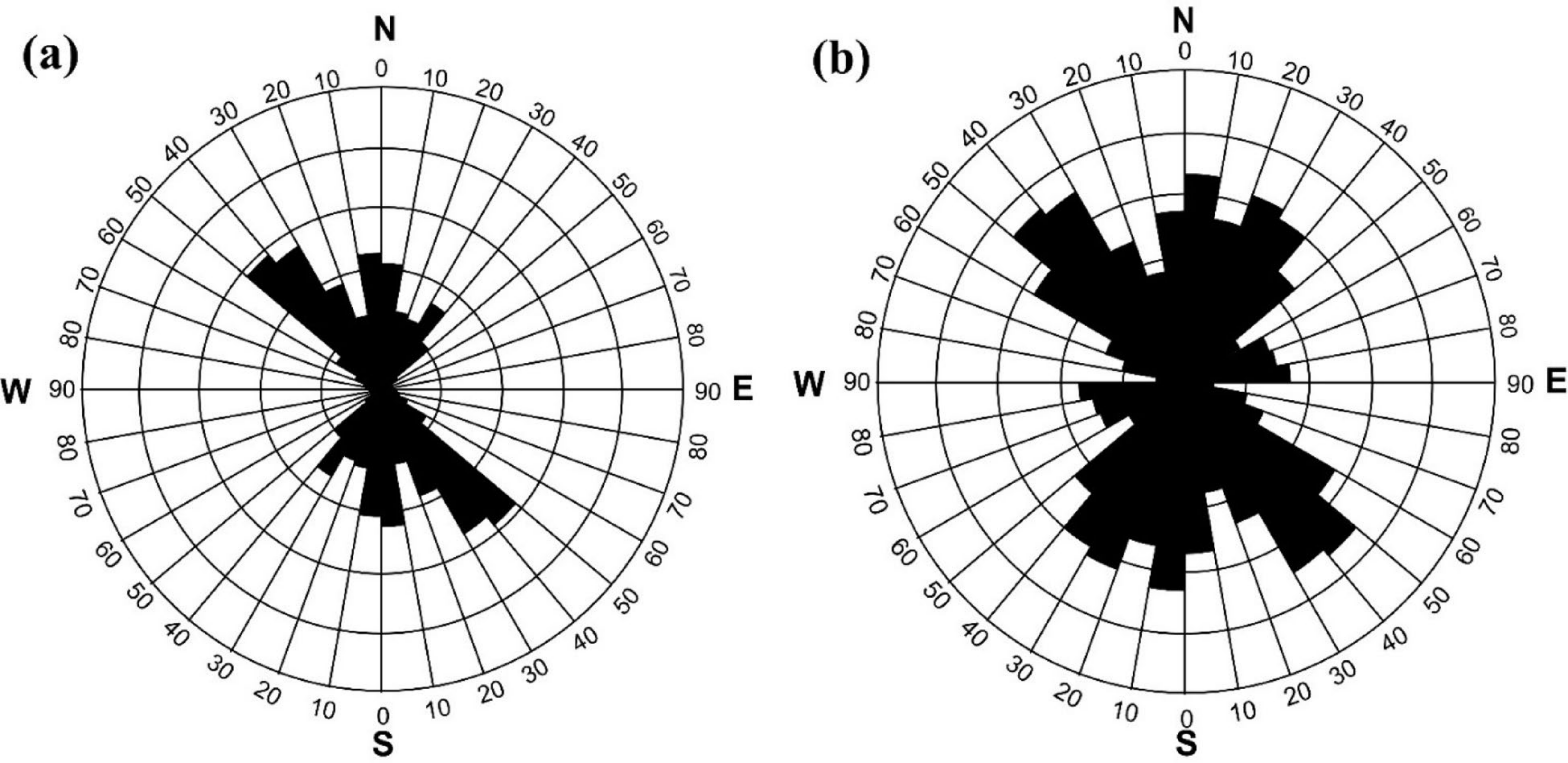
Rose diagram of; **a**) Small-scale lineaments; and **b**) Large-scale lineaments.

As above-mentioned, the MDA technique is applied to the RTP magnetic anomaly (Fig. 2) giving two different maps at small- and large-scale (Fig. 3). In the small-scale MDA-EHD map (Fig. 3a), the detailed lineaments of the shallower depths’ structures are shown while the large-scale magnetic MDA-EHD map (Fig. 3b) defines the regional structures in Wadi Dif area. Figure 4 illustrates the lineaments that were extracted from the MDA-EHD maps in Fig. 3. Tables 3 and 4 show the main parameters of the major fault trends detected from MDA-EHD magnetic maps in Fig. 4a and b, respectively. These fault trends and lineaments were statistically analyzed via rose diagram (Fig. 5).

**Table 3:** The main parameters of the major fault trends detected from small-scale MDA-EHD magnetic map.

WEST					Azimuth	EAST				
*N*	*N*%	L	L%	L/*N*		L/*N*	L%	L	*N*%	*N*
1102.0	55.2	75474.4	10.7	68.5	0:<10	857.8	10.2	72057.8	4.2	84.0
60.0	3.0	41190.3	5.8	686.5	10:<20	737.8	6.9	48693.0	3.3	66.0
91.0	4.6	67644.3	9.6	743.3	20:<30	695.0	6.8	47954.1	3.5	69.0
128.0	6.4	97458.9	13.8	761.4	30:<40	644.4	8.3	58641.9	4.6	91.0
130.0	6.5	102007.5	14.4	784.7	40:<50	594.4	5.3	37449.4	3.2	63.0
52.0	2.6	29460.4	4.2	566.5	50:<60	563.1	1.7	11825.8	1.1	21.0
12.0	0.6	6182.5	0.9	784.7	60:<70	385.8	0.4	2700.6	0.4	7.0
6.0	0.3	2068.0	0.3	761.4	70:<80	341.1	0.3	2387.9	0.4	7.0
4.0	0.2	1628.2	0.2	743.3	80:<90	570.0	0.2	1140.1	0.1	2.0
1585.0	79.4	423114.4	59.9		Sum		40.1	282850.4	20.6	410.0
∑n	1995.0		∑L	705964.9		∑n%	100.0		∑L%	100.0

**Table 4:** The main parameters of the major fault trends detected from large-scale MDA-EHD magnetic map.

WEST					Azimuth	EAST				
*N*	*N*%	L	L%	L/*N*		L/*N*	L%	L	*N*%	*N*
18.0	6.4	43072.5	7.0	2392.9	0:<10	2277.5	8.1	50105.5	7.8	22.0
12.0	4.3	28693.9	4.6	2391.2	10:<20	2040.3	6.6	40806.0	7.1	20.0
17.0	6.0	37197.7	6.0	2188.1	20:<30	2528.9	7.8	48048.3	6.8	19.0
22.0	7.8	54379.8	8.8	2471.8	30:<40	1928.6	7.5	46286.4	8.5	24.0
20.0	7.1	54482.6	8.8	2724.1	40:<50	2136.3	5.9	36317.6	6.0	17.0
24.0	8.5	43361.3	7.0	1806.7	50:<60	2651.3	2.6	15907.9	2.1	6.0
12.0	4.3	18865.8	3.1	2724.1	60:<70	2620.0	3.8	23579.6	3.2	9.0
10.0	3.6	16282.5	2.6	2471.8	70:<80	2014.3	3.9	24171.1	4.3	12.0
4.0	1.4	8348.5	1.4	2188.1	80:<90	2103.3	4.4	27342.6	4.6	13.0
139.0	49.5	304684.6	49.4		Sum		50.6	312565.1	50.5	142.0
∑n	281.0		∑L	617249.7		∑n%	100.0		∑L%	100.0

Figures 3 and 4, and 5 clearly illustrate that the shallow (small-scale) lineaments of Wadi Dif are directed in the NW, N-S, NE, NNE, and NNW directions. While the main regional (large-scale) lineaments prevailing the area are NW, N-S, NE, NNE, NNW, and E-W directions.

### Remote sensing

RS data were utilized to enhance geological mapping and mineral exploration in the Wadi Dif area. Landsat-8 and ASTER imagery facilitated lithological discrimination and identification of hydrothermal alteration zones through techniques like PCA, band ratios, and Constrained Energy Minimization (CEM). Additionally, Sentinel-1B radar data were used for automated lineament extraction, revealing dominant structural trends that influence mineralization.

#### Lithological mapping utilizing Landsat-8

PCA is a widely utilized multivariate statistical method that employs an orthogonal transformation to convert a collection of correlated variables into a set of linearly independent variables. The current study has specifically focused on the basement units (e.g., gneiss, metasediments, metavolcanics, syn and late granitoids), which are considered to be prospective host rocks for hydrothermal mineral deposits, including gold, silver, and copper. Various colored composites enclosing Decorrelated FCC 567, Abrams’s BR 6/7 4/3 5/6, Kaufmann’s BR 7/5 5/4 6/7 and PC 213 in RGB have been employed for lithological discrimination, facilitating the identification of major structural features and the updating of existing geological maps for the study area.

**Fig. 6:**
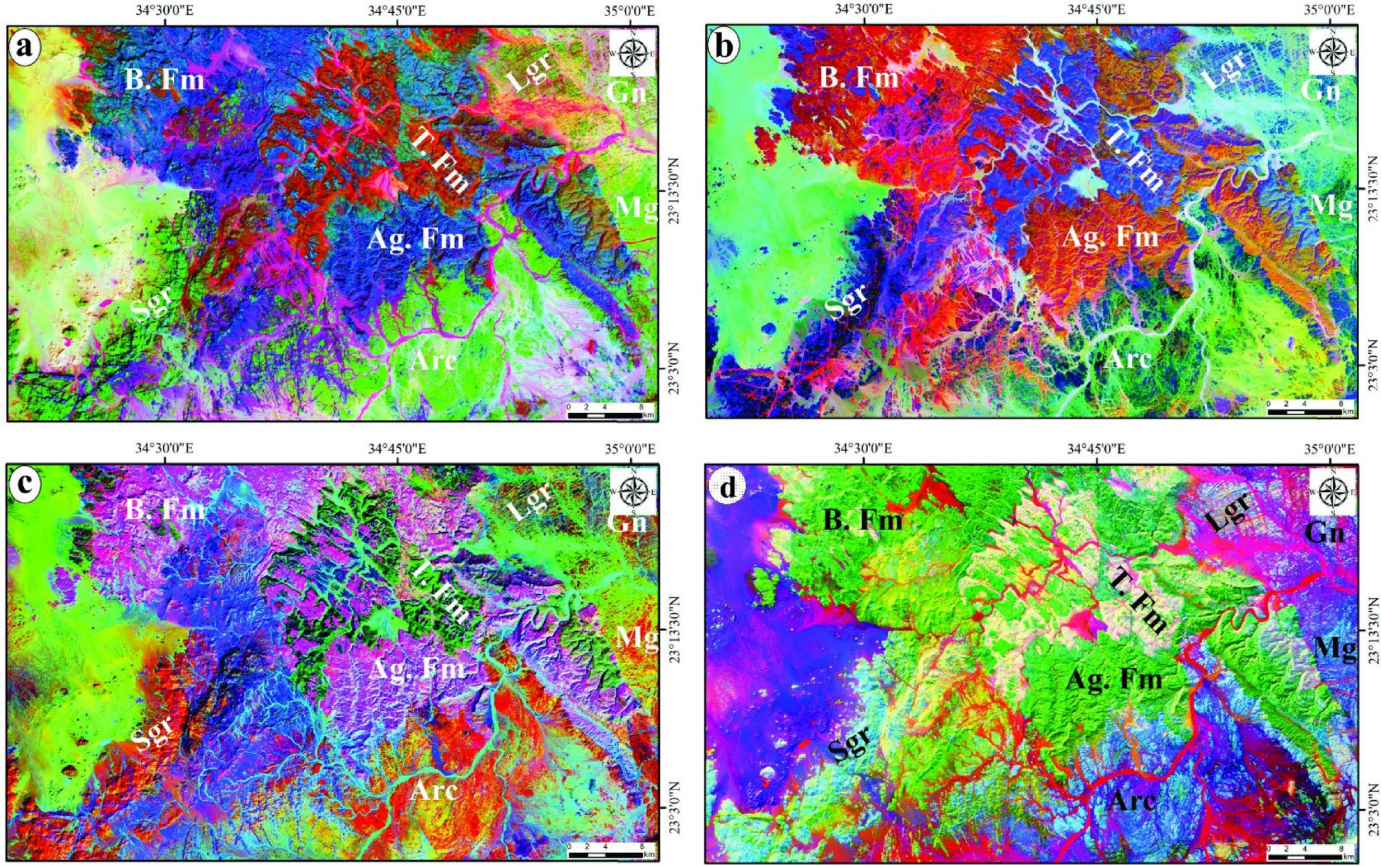
Lithological discrimination via Landsat-8; **a**) Decorrelated FCC 567; **b**) Abrams’s BR 6/7 4/3 5/6, **c**) Kaufmann’s BR 7/5 5/4 6/7 and **d**) PC 213 in RGB. Abbreviation: Abu Aggag (Ag. Fm), Timsah (T. Fm) and Um Bramil (B. Fm), gneiss (Gn), arc assemblages (Arc), metagabbro (Mg), syn-tectonic granite (Sgr) and late-tectonic granite (Lgr). (The figure was created by ENVI v. 5.6.2. software; (https://www.l3harrisgeospatial.com/Software-Technology/ENVI)).

Decorrelated FCC 567-RGB (Fig. 6a) discriminated successfully between the lithological contacts of the three cretaceous formations (Fm) which have appeared in pale blue, reddish orange and deep blue tones for Abu Aggag (Ag. Fm), Timsah (T. Fm) and Um Bramil (B. Fm) respectively and basement rocks highlighted by apple green, cyan-green, yellowish green, shiny green, and brownish green for gneiss, arc assemblages (metasediments and metavolcanics), metagabbro, syn-tectonic granite and late-tectonic granite respectively. Both band ratios of Abrams’s BR 6/7 4/3 5/6 and Kaufmann’s BR 7/5 5/4 6/7 in RGB mode were useful to highlight the basement rocks from the cretaceous formations (Figs. 6b and c). In Abrams’s BR 6/7 4/3 5/6-RGB (Fig. 6b), the Precambrian units were distinguished by cyan, greenish blue, greenish cyan, deep blue and pale blue colors for gneiss, arc assemblages, metagabbro, syn-tectonic granite and late-tectonic granite respectively, while the cretaceous formations were discriminated by orange, violet-blue and reddish orange colors for Ag Fm, T. Fm and B. Fm respectively. The appearance of the basement rocks varies from pale orange to deep orange tones while the cretaceous formations were characterized by purple and green tones in Kaufmann’s BR 7/5 5/4 6/7-RGB (Fig. 6c). The contacts between the sedimentary rocks and hard rocks were detected also using PC 213-RGB (Fig. 6d) where the grading of blue color emphasized the basement rocks as bluish white for gneiss and late-granite, light blue for the arc units, cyan-blue for the metagabbro and shiny cyan for the syn-granitoids.

#### Delineation of hydrothermal alteration zones using optical satellite imagery

**Fig. 7:**
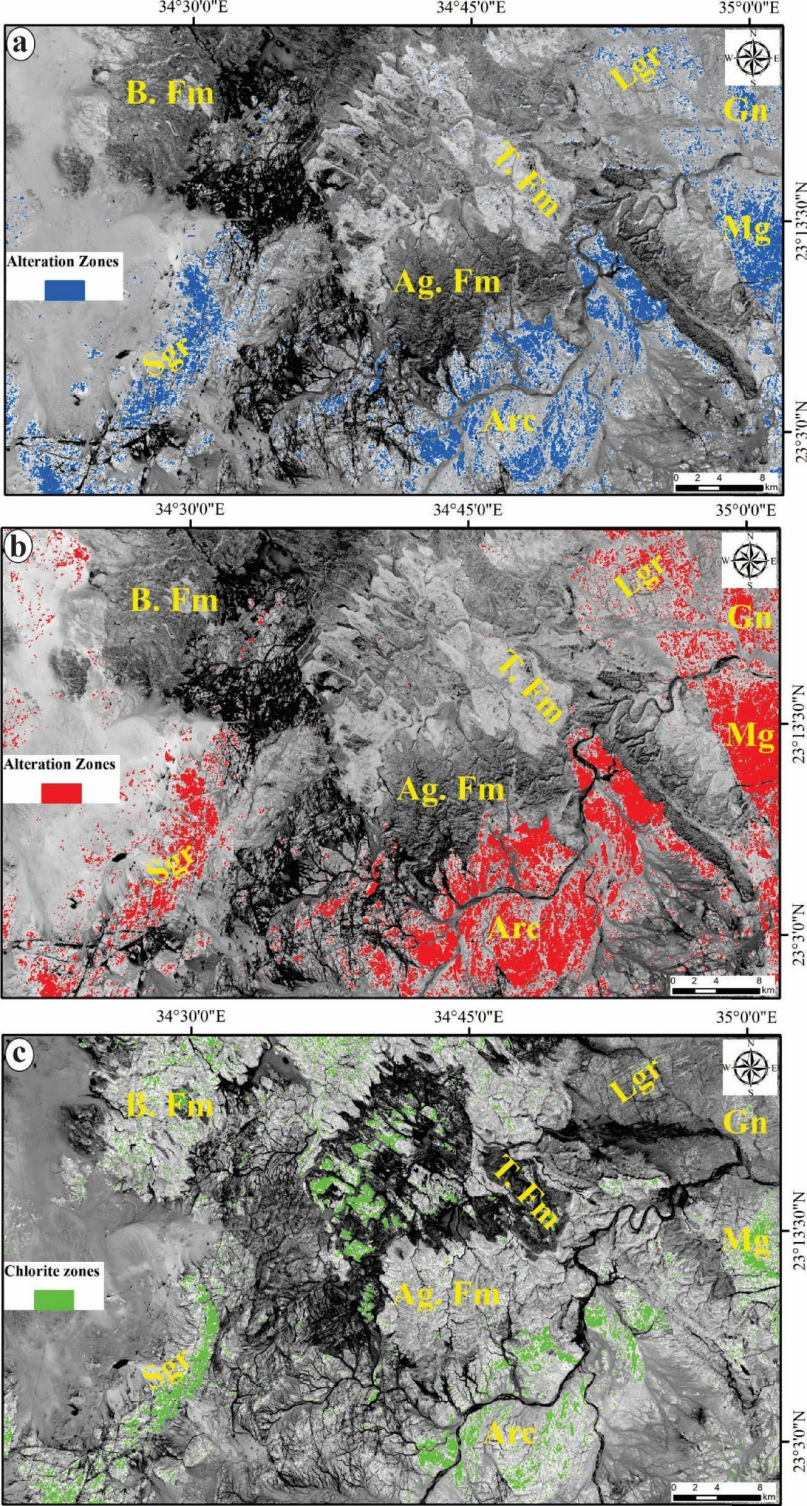
Identification of the alteration zones via Landsat-8: **a**) BR 6/7 and **b**) PC3 for CO3 and Mg-OH bearing minerals and **c**) BR 7/5 for chlorite zones in the grey-scale. For abbreviation see Fig. 1. (The figure Created by ArcGIS Desktop v. 10.8. https://www.esri.com/en-us/arcgis/products/arcgis-desktop/overview).

**Fig. 8:**
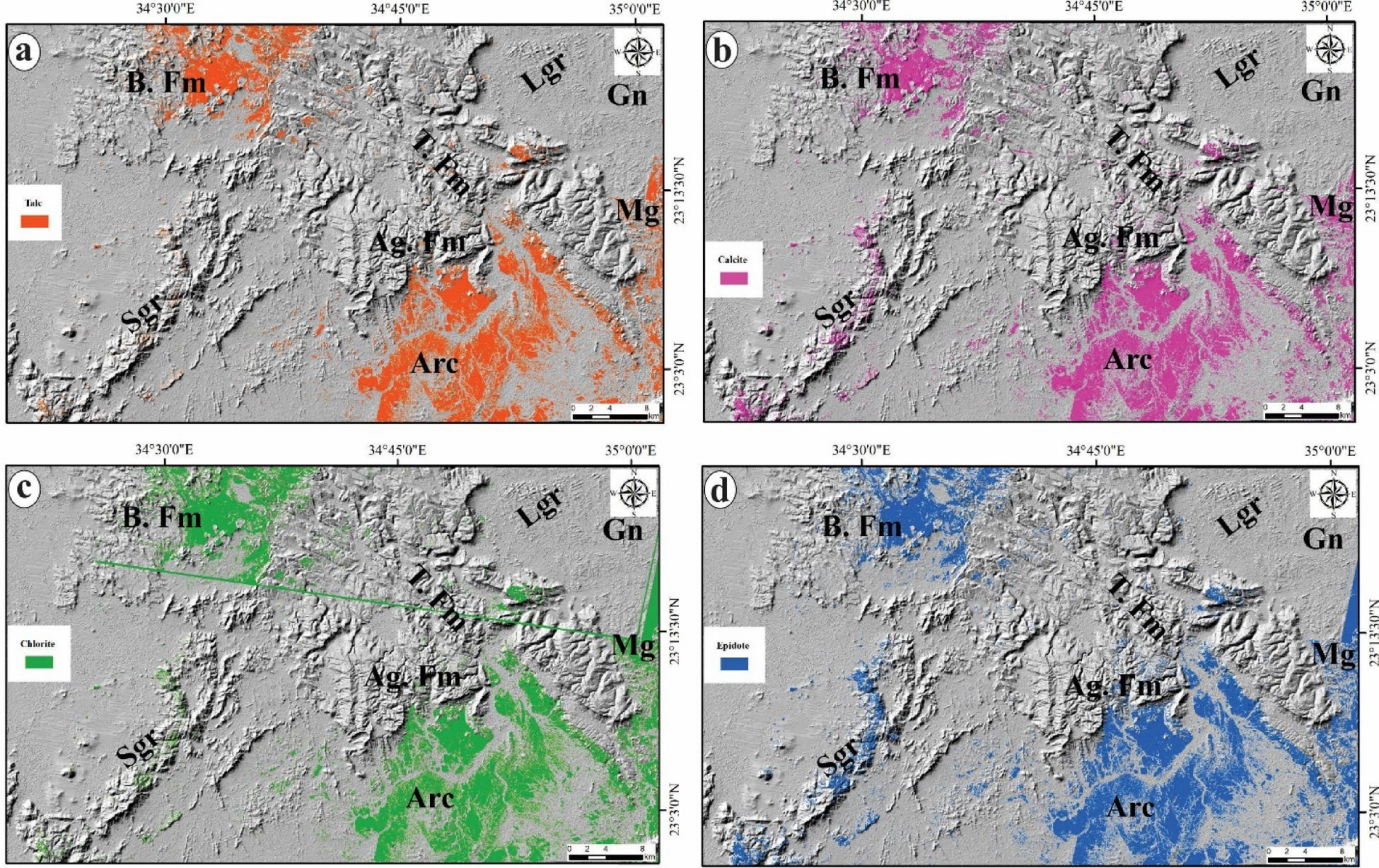
Surface distribution of the four alteration minerals of propylitic zones via CEM technique applied on ASTER data: **a**) Talc, **b**) Calcite, **c**) Chlorite and **d**) Epidote alteration minerals. For abbreviation see Fig. 1. (The figure Created by ArcGIS Desktop v. 10.8. https://www.esri.com/en-us/arcgis/products/arcgis-desktop/overview).

Hydrothermal alteration zones are widely recognized as highly conducive environments for the formation and occurrence of ore deposits. Consequently, the identification and mapping of these alteration zones are essential for pinpointing areas likely to contain associated ore deposits in the study area. The constrained energy minimization (CEM) method is recognized as a technique for spectral mapping. It seeks to enhance the spectral response of a target by diminishing the influence of all other features, which are regarded as an unknown background. To support this procedure, optical data sourced from Landsat-8 and ASTER have been utilized. Specifically, the band ratios of 6/7 and 7/5 as well as PC3 in the grey-scale imagery of Landsat-8 (Figs. 7a, b and c), along with the Constrained Energy Minimization (CEM) technique applied to the initial nine VNIR + SWIR bands of the ASTER data to allocate the calcite, talc chlorite and epidote minerals (Figs. 7a-c) which are characterized the propylitic zones (Fig. 8). Through the manipulation of the rule threshold (Table 5) using the CEM method and guided by the spectral library from USGS, grey-scale images were created to highlight the surface distribution of the four alteration minerals as a color coding providing a clearer understanding of their respective locations.

**Table 5:** Statistical overview summarizing the CEM method utilized for the alteration of minerals.

Alteration Zone	Mineral	Method	Rule Threshold	Target Count	Average Area km^2^
Propylitic zone	Calcite	CEM	0.130	21,877	12142.513
	Talc		0.130	19,836	13426.055
	Chlorite		0.130	21,832	15829.624
	Epidote		0.130	22,779	14930.383

#### S1B based lineaments extraction

**Fig. 9:**
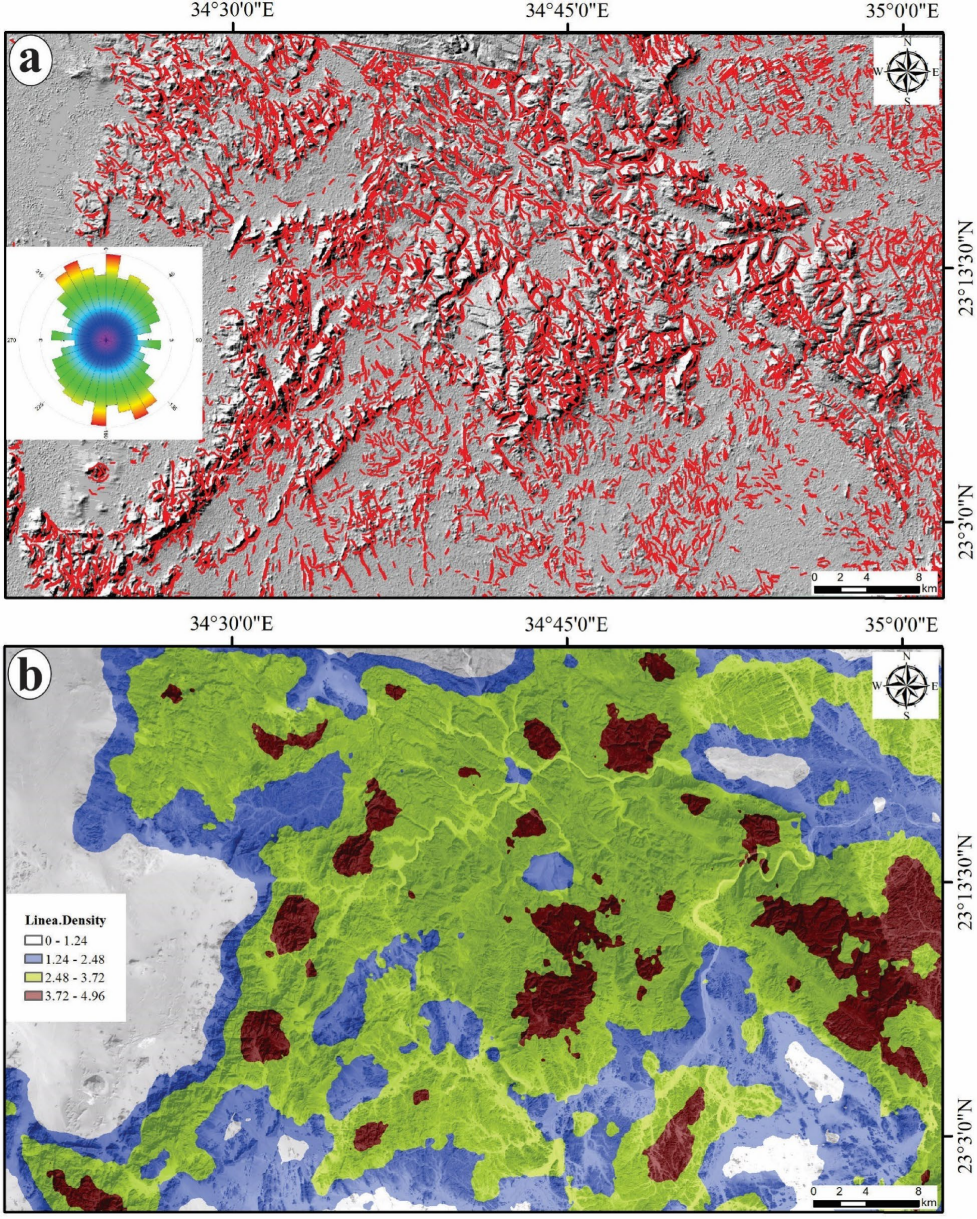
Automatic lineament extraction via S1B: **a**) Hillshaded lineament map and **b**) Lineament Density map. (The figure Created by ArcGIS Desktop v. 10.8. https://www.esri.com/en-us/arcgis/products/arcgis-desktop/overview).

The identification of prevailing tectonic trends and the delineation of potential pathways for the movement and deposition of ore-bearing hydrothermal fluids within a given region can be achieved through the extraction of lineaments. The principal components (PCs1) derived from the analysis of S1B radar data have been utilized for the automated extraction of lineaments (Fig. 9a). This process has resulted in the creation of lineament maps that illustrate the surface distribution of various geological features, such as faults, joints, and dykes, represented as short, dense red lines, along with azimuth rose diagrams generated using Rockwork software for the two specified areas (Fig. 9a). Additionally, a lineament density map has been developed based on the surface distribution of the extracted lineaments, highlighting the concentrations of these features (Fig. 9b). According to the azimuth frequency diagrams of the investigated area, the dominant trends of the lineaments/fracturs in the study area are predominantly aligned in the following decreasing order N-S, NW-SE, NE-SW and E-W (Fig. 9a).

### Structural setting

The Beitan gneiss complex is characterized by a distinctive arrangement of antiforms and synforms that align with the NNW-SSE orientation of the Beitan shear belt^4,62,79^. This shear belt encompasses extensive regions exhibiting sinistral shearing. It represents the southeastern extension of the Kharit-Hodein shear zone, a significant subvertical, NW-trending strike-slip shear zone in the SED, extending approximately 186 km^4^. The Beitan shear belt is marked by brittle-ductile deformation and trends NNW with a steep dip. It is bordered by a thrust fault boundary, which delineates the base of an overthrust sheet. The Beitan gneiss complex comprises well-defined gneissic bands of varying thickness, consisting of extensively deformed mineral aggregates such as mica, quartz, feldspar, and amphibole. Metasedimentary rocks and amphibolite display a broad foliation characterized by compositional stratification and the preferential alignment of mineral grains. The structural and textural characteristics indicate that the foliation in the gneiss and gneissose granite originated from a magmatic structure, which subsequently experienced deformation, resulting in a flat, planar fabric. The banded amphibolite exhibits foliation with light-gray bands interspersed with darker layers of well-aligned and significantly deformed amphibole, feldspar, and chlorite.

The Beitan gneiss complex is characterized by a distinct NNW-oriented fabric, featuring folds that vary from open to recumbent forms. These folds encompass both overturned and recumbent geometries, with steep limbs that exhibit a moderate dip toward the southwest or are oriented vertically in the case of overturned structures. Adjacent to these folds, the granite gneiss displays a moderate dip toward the northeast, which gradually becomes less pronounced as one moves southward, resulting in a flat limb. The axial trace of the north-trending F2 antiform is located near the boundary between the gneiss and the supracrustal materials.

Schists exhibit distinct schistose and mylonitic foliation characteristics. The structural fabric is marked by moderately to steeply dipping foliations, with strike orientations ranging from NW-SE to N-S. The initial foliation (S1) and associated folds (F1) underwent deformation into NW-trending folds (F2), which are characterized by S2 axial planar foliation. Subsequently, these NW-oriented F2 folds were further deformed into N- and NE-trending folds (F3), displaying S3 axial planar foliation. Notably, there is a slight dominance of S3 axial planar foliations within high-strain zones, which are delineated by kink bands or spaced cleavages that align with the axial planes of the N-trending folds (S3). Kink bands represent sharp deviations of S2. In the Beitan gneiss belt, high-strain zones featuring subvertical foliation planes are generally parallel and oriented NNW–SSW, exhibiting isoclinal and intrafolial folds with shallow plunging fold hinges and subvertical axial planar foliation. Additionally, shallow NNW- or SSE-plunging mineral and stretching lineations are observed on the steep foliation planes within the NNW–SSE-striking high-strain zones.

The region has experienced an extended period of brittle deformation linked to the intrusion and emplacement of late-orogenic granites, as well as the exhumation of terranes. During this time, the area has been intersected by numerous dextral strike-slip faults that impact entire rock units, including the post-orogenic granites and previously formed structures. The Cretaceous formations within the Kharit basin are situated in an elongated graben, which is bordered by rift faults that extend in a northwest-southeast direction^80,81^ and dip toward the northeast and southwest. Four distinct fault populations with varying orientations have been identified: northwest, west-northwest, north-northeast to south-southwest, and east-west. These populations exhibit differing degrees of dip and strike-slip components, aligned with the primary extension direction. The north-south trending faults predominantly exhibit left-lateral strike-slip characteristics, displacing all other fault populations. These north-south faults extend for approximately ten kilometers and consist of multiple segments arranged in a right-stepping pattern. They intersect and sinistrally offset the northwest and east-west oriented faults. The northwest and west-northwest faults are interconnected along their lengths, forming the rift-bounded and rift-interior fault systems associated with the Cretaceous rocks of the Khatit basin. Areas of intersection are significantly deformed and characterized by a complex fracture pattern. The slickensides observed on the north-south trending faults are oblique, suggesting a strike-slip movement with a minor dip-slip component.

The deformation history of the region under investigation initiates with an early NNE–SSW crustal shortening associated with arc–arc collision (D1), which is evidenced by the formation of S1 foliations and WNW-trending F1 early folds observed in gneisses and schists. The subsequent phase, D2, is characterized by NNW–SSE folds and NNW–SSE crenulation cleavage (S2). This phase involved sinistral transpression along NNW–SSE ductile shear zones, resulting from NW-ward nappe stacking and thrusting. The N–S to NE-SW crenulations and kink folds (F3) likely emerged from oblique non-coaxial deformation of the cleaved rocks. The fourth deformation event, D4, was marked by brittle deformation, which led to the formation of the Kharit graben and the deposition of Cretaceous sediments, followed by the development of ENE–WSW dextral and N-S sinistral strike–slip faults that further altered the preexisting rocks and displaced earlier structures.

## Discussion

### Aeromagnetic data analysis

The MDA-EHD maxima defines very well the boundaries of the magnetic anomalies’ causative sources^82^. Additionally, due to its definition, the EHD can be used to investigate geological structures of diverse depths and/or extensions, with a suitable choice of weights and differentiation order. This is crucial to get different images of the boundaries of the magnetic causative source. Consequently, this leads to a multiscale derivative analysis exhibiting the boundaries at large and small scales^71^. A further benefit of MDA technique is that it performs similarly to a filtering process, without applying a separation of potential field anomaly components.

### Lithological, structural and alteration mapping

The disparities in tonal characteristics among the identified color composites, specifically FFC, BRs, and PCA, throughout the region of interest were instrumental in accentuating lithological contacts. This study permitted the identification of major structural elements such as thrusts, strike-slip faults, and folds, thereby updating the geological and structural maps (See the geological map). Decorrelated FCC 567, Abrams’s BR 6/7 4/3 5/6, Kaufmann’s BR 7/5 5/4 6/7 and PC 213 in RGB mode were able to highlight the sharp contacts between the Cretaceous formations and basement rocks, besides they enable us to monitoring joints, the major normal and strike-slip faults as well as folds which were recorded within the arc assemblages at the southern sector of the study area (Fig. 1b). Abrams’s BR 6/7 4/3 5/6-RGB was the most efficient ratio which successfully differentiated between the lithological units of basement rocks recorded at the eastern, southern and southwestern parts of the presented area. Moreover, the three Cretaceous formations were more detectable though the PC 213 (Fig. 2d).

The band ratios of 6/7 and 7/5 as well as PC3 in the grey-scale imagery of Landsat-8 (Figs. 6a, b and c) were helpful to allocate the general alteration zones and show their surface distribution which are concentrated over the basement rocks. Br 6/7 and PC3 (Figs. 7a and b) utilized to detect the areas enriched by CO_3_ and Mg-OH bearing minerals and it noticed that all the recorded areas of these minerals were overlapped with the areas of basement rocks. Also, Br 7/5 is employed to highlight the chlorite zones which are recognized mainly in metagabbro, arc assemblages and syn-tectonic granite as well as some parts within Timsah formation (Fig. 7c).

**Fig. 10:**
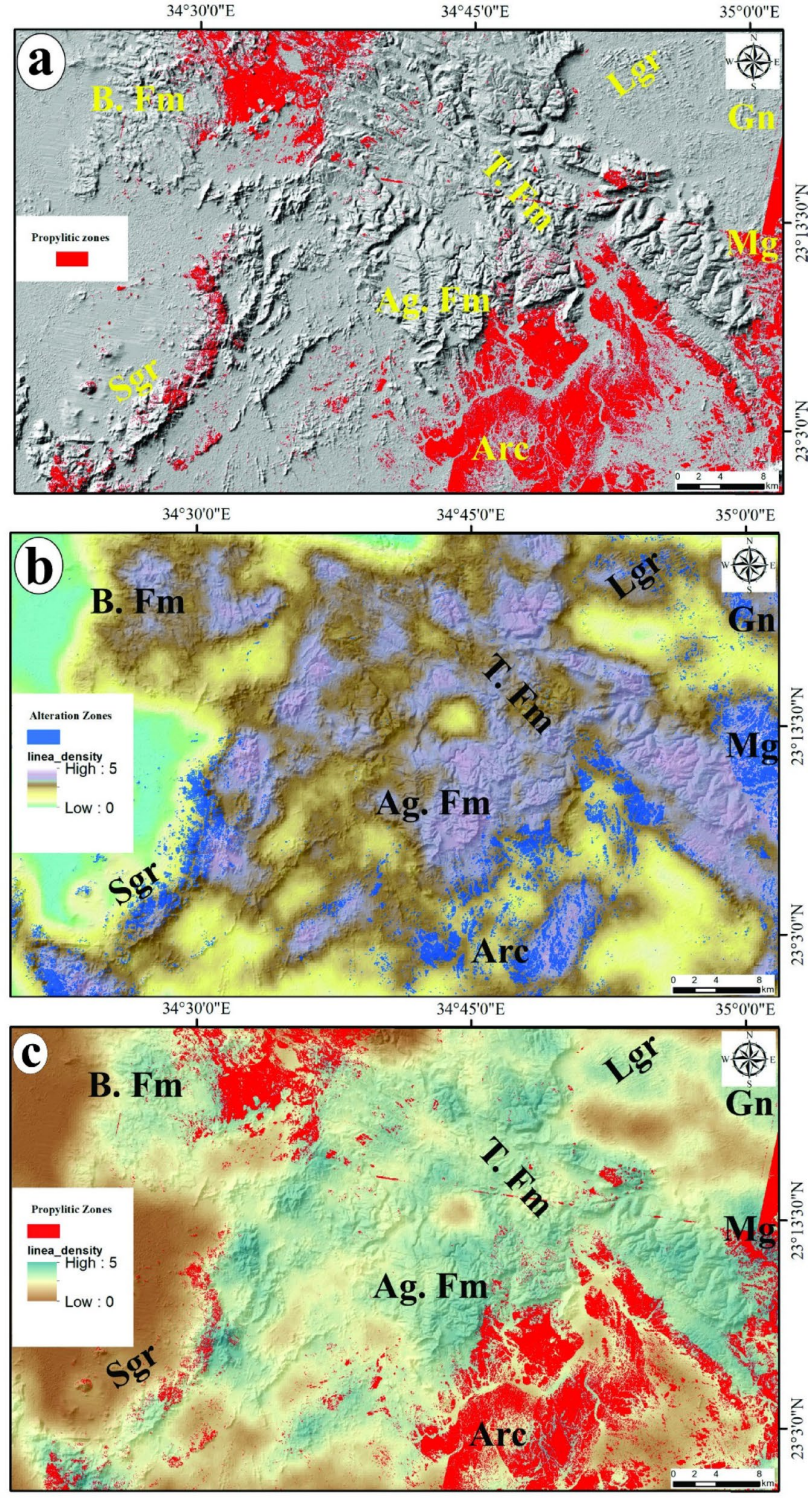
**a**) surface distribution of the propylitic zones, **b**) and **c**) The spatial distribution of alteration zones as identified by Landsat-8 and ASTER in comparison to the Lineament Density map. For abbreviation see Fig. 1. (The figure Created by ArcGIS Desktop v. 10.8. https://www.esri.com/en-us/arcgis/products/arcgis-desktop/overview).

The constrained energy minimization (CEM) technique is classified as a spectral mapping method, as articulated by Harsanyi et al.^83^. This technique is predicated on the surface reflectance data obtained through remote sensing, in conjunction with reference spectra of the minerals found in the target al.teration zones, which can be derived from spectral analyses or spectral libraries. The application of CEM in supervised classification of ASTER data has proven effective in mapping the spatial distribution of four principal alteration minerals: chlorite, epidote, calcite, and talc, which are characteristic of propylitic zones. The spatial distribution of the four alteration minerals, as illustrated in Figs. 7a-c, identified through the CEM method which are represented the propylitic zones depicted in Fig. 10a corresponds with the alteration sites identified by Landsat-8 (Figs. 7a-c). Upon comparing the propylitic zones identified by ASTER with those enriched in CO3 and Mg-OH minerals allocated by Landsat-8, it becomes evident that they encompass identical areas characterized by granitic, metagabbro, and arc assemblages which are recognized as significant sources for gold mineralization and other related ore deposits.

The Beitan gneiss complex is a unique geological formation that aligns with the NNW-SSE orientation of the Beitan shear belt. It is the southeastern extension of the Kharit-Hodein shear zone, a significant subvertical, NW-trending strike-slip shear zone in the SED. The complex comprises well-defined gneissic bands of varying thickness, consisting of extensively deformed mineral aggregates such as mica, quartz, feldspar, and amphibole. The gneiss and gneissose granite display a broad foliation characterized by compositional stratification and the preferential alignment of mineral grains. The Beitan gneiss complex is characterized by a distinct NNW-oriented fabric, featuring folds that vary from open to recumbent forms. The structural fabric is marked by moderately to steeply dipping foliations, with strike orientations ranging from NW-SE to N-S. The region has experienced an extended period of brittle deformation linked to the intrusion and emplacement of late-orogenic granites and the exhumation of terranes. Schists have distinct schistose and mylonitic foliation characteristics, with moderately to steeply dipping foliations and strike orientations ranging from NW-SE to N-S. The initial foliation deformed into NW-trending folds, followed by N- and NE-trending folds, with S3 axial planar foliations dominating high-strain zones. In the Beitan gneiss belt, high-strain zones have subvertical foliation planes and isoclinal folds. The Cretaceous formations within the Kharit basin are situated in an elongated graben, bordered by rift faults that extend in a northwest-southeast direction. Four distinct fault populations with varying orientations have been identified: northwest, west-northwest, north-northeast to south-southwest, and east-west. The deformation history of the region begins with an early NNE–SSW crustal shortening associated with arc–arc collision (D1). The subsequent phase, D2, is characterized by NNW–SSE folds and NNW–SSE crenulation cleavage (S2). The fourth deformation event, D4, was marked by brittle deformation, leading to the formation of the Kharit graben and the deposition of Cretaceous sediments.

### Integration of alteration zones to lineament density

The hydrothermal minerals found in alteration zones, such as carbonate and minerals containing Fe and Mg-OH, are indicative of the alteration processes affecting basic-ultrabasic and granitic rocks. These minerals exhibit unique spectral characteristics, as noted by Harsanyi et al.^83^. The alteration minerals can be categorized into four primary zones: (i) the argillic zone, which comprises kaolinite, illite, alunite, and montmorillonite; (ii) the phyllic zone, characterized by illite and muscovite; (iii) the propylitic zone, which includes chlorite, epidote, calcite, and talc; and (iv) the gossan zone, consisting of hematite, goethite, and jarosite. The presented study focused on the propylitic zones, CO_3_ and Mg-OH bearing minerals which are prevalent in the majority of Precambrian rocks. We deliberately excluded the other two zones of clay minerals, as they may be influenced by the presence of Cretaceous formations that could be enriched with clays.

The lineaments and fractures are considered effective conduits and suitable pathways for the ascending hydrothermal solutions enriched by several types of minerals/ores. Moreover, the areas with high concentrations of lineaments have high potentiality for the flowing of the alteration minerals and ore deposits accompanied with the hot fluids. So, the lineaments, particularly the areas of high lineament density, are one of the most favorable areas where these hot fluids act with the country rocks causing the perception of the alteration minerals and their related ore deposits.

A high density of lineaments may suggest an increased extent of rock fracturing, which is often correlated with mineralization processes. The potential for gold mineralization is heightened in areas where there is a significant density of lineaments and fractures associated with hydrothermal alteration zones. Consequently, the lineament density map (Fig. 9b) of the study area has been overlaid with the alteration zones (Figs. 5c, 6a and 9b) which have been accurately delineated using the band ratios and PC of Landsat-8, besides the CEM method applied on ASTER data. The spatial correlation between the alteration zones and surface distribution of lineament density (Fig. 10b and c) exhibiting exceptionally high lineament densities underscores the importance of these features in the mineralization process. A thorough analysis of this map may reveal the most probable mineralization zones within the study area.

The distribution patterns of lineament density and surface alteration zones have facilitated the development of two potential maps (Fig. 10b and c) for the designated areas, highlighting their interconnections and pinpointing regions with a high probability of ore deposits linked to alteration minerals. The analysis reveals that the identified alteration zones are primarily situated in areas with moderate to high lineament density, which allows for the efficient movement of hydrothermal fluids and the deposition of ores. However, certain regions within the investigated area display high lineament density but lack to the alteration minerals or zones. This observation raises the possibility that the absence of alteration may stem from differences in rock composition or that these high-density lineament areas were not significantly influenced by hydrothermal fluids, resulting in no alteration minerals being present. Additionally, it has been previously established that the dominant trend of these lineaments are mainly the N-S and NW northwest coinciding with the N-S Hamisana shear zone (HSZ) and northwest-trending Najd fault system (NFS)^5,16^. Therefore, it can be inferred that the majority of lineaments, which are deemed favorable pathways for ore-bearing fluids in the study area have been formed and influenced by the HSZ and NFS. The controlling of HSZ and NFS in the mineralization of ores in Dif area has been supported by the overlapping of the high percentage of identified alterations minerals at the sectors of high density of linear structures (Fig. 10) which striking mainly N-S and NW according to the azimuth diagram (Fig. 9a) coinciding with the tectonic trends of both shear zones. This finding proved that the linear structures including faults, fractures and joints developed during the activations of HSZ and NFS have formed the crucial pathways for the ore mineralization in the presented area.

Finally, integrating the aeromagnetic data analysis with RS and geological data in our present study has provided a detailed framework for comprehending the tectonic pattern, mineral potential, and deep-seated and shallow structural architecture. Aeromagnetic data analyses have been integrated with surface features recognized through RS, enriching the accuracy of unveiling deep-seated faults, structural mapping, and deformation systems. This multi-disciplinary strategy has significantly enhanced the reliability of subsurface analyses. The integrated investigation has not only refined the revealing of structural edges but also underlined key zones with possible mineralization, documenting the significance of integrated methodologies in analyzing complicated and challenging geological terrains.

## Conclusion

Geological mapping in remote and rugged terrains, such as Wadi Dif in the South Eastern Desert of Egypt, faces significant challenges due to limited accessibility and scarce field data. To address these issues, this study integrated aeromagnetic and remote sensing data, which effectively enhanced the identification of structural features and alteration zones in these hard-to-reach areas.

Multiscale derivative analysis of aeromagnetic data has revealed both shallow and small-scale lineaments trending mainly in the NW, N-S, NE, NNE and NNW directions and deep-seated and large-scale lineaments prevailing in NW, N-S, NE, NNE, NNW and E-W directions, reflecting a complex deformation history.The deformation history of the area starts with a primary phase of crustal shortening oriented NNE–SSW, resulting in arc-arc collision. This is supported by S1 foliations and WNW-trending F1 early folds. The second phase, D2, involves NNW–SSE folds and crenulation cleavage, resulting from NW-directed nappe stacking and thrusting. The fourth episode, D4, is marked by brittle deformation, leading to the Kharit graben and Cretaceous deposits. This is followed by the formation of ENE–WSW dextral and N-S sinistral strike-slip faults.

Remote sensing analysis, using constrained energy minimization (CEM) technique successfully maps four main alteration minerals: chlorite, epidote, calcite, and talc, linked to propylitic zones. There is a connection between these alteration minerals and the alteration areas identified from Landsat-8and they showed high agreement in areas known for gold mineralization and related ore deposits.

The spatial correlation between surface alteration zones and areas of moderate to high lineament density highlights the structural control on hydrothermal fluid movement and ore deposition. The main trends of these lineaments follow the N-S and NW directions, aligning with the Hamisana shear zone and the Najd fault system. Thus, most lineaments, considered good pathways for ore fluids, are shaped by these zones.

Our results demonstrate that the success of this investigation highlights the importance of integrating remote sensing techniques and multiscale derivative analysis (MDA) of aeromagnetic data in determining structural complexities and delineating zones of mineral potential, particularly in provinces with unreasonable accessibility. By analyzing multiple datasets and employing various strategies, we conducted a more comprehensive interpretation of the subsurface setting than would be possible via a single technique alone. This multidisciplinary approach not only enhanced the reliability of our understanding but also revealed its adaptability and usefulness in comparable geologically complicated regions. Such a strategy holds influential promises for guiding forthcoming exploration ambitions in underexplored and remote areas globally.

## Data Availability

Data sets generated during the current study are available from the corresponding author on reasonable request.
